# Diagnostic challenges of autism spectrum disorder in women without intellectual or language impairments: a narrative review

**DOI:** 10.25122/jml-2025-0118

**Published:** 2025-08

**Authors:** Alexandra Dolfi, Cătălina Tudose

**Affiliations:** 1 Prof. Dr. Alexandru Obregia, Psychiatry Hospital, Bucharest, Romania; 2 Doctoral School, Carol Davila University of Medicine and Pharmacy, Bucharest, Romania; 3 Department of Psychiatry, Carol Davila University of Medicine and Pharmacy, Bucharest, Romania

**Keywords:** autism spectrum disorder (ASD), high-functioning autism (HFA), female phenotype, delayed diagnosis, cognition disorders/diagnosis, language development disorder/diagnosis, ADHD, Attention-Deficit Hyperactivity Disorder, ADI-R, Autism Diagnostic Interview-Revised, ADOS-2, Autism Diagnostic Observation Schedule, Second Edition, AQ, Autism Quotient, AS, Asperger Syndrome, ASD, Autism Spectrum Disorder, BASC-3, Behavior Assessment System for Children, Third Edition, BPD, Borderline Personality Disorder, CAT-Q, Camouflaging Autistic Traits Questionnaire, DSM-5, Diagnostic and Statistical Manual of Mental Disorders, Fifth Edition, DSM-5-TR, Diagnostic and Statistical Manual of Mental Disorders, Fifth Edition, Text Revision, DSM-IV, Diagnostic and Statistical Manual of Mental Disorders, Fourth Edition, DSM-IV-TR, Diagnostic and Statistical Manual of Mental Disorders, Fourth Edition, Text Revision, DSM-III, Diagnostic and Statistical Manual of Mental Disorders, Third Edition, DSM-III-R, Diagnostic and Statistical Manual of Mental Disorders, Third Edition Revised, GQ-ASC, Girls Questionnaire for Autism Spectrum Condition, HFA, High-Functioning Autism, ICD, International Classification of Diseases, OCD, Obsessive-Compulsive Disorder, PTSD, Post-Traumatic Stress Disorder, RAADS-R, Ritvo Asperger and Autism Diagnostic Scale-Revised, SfA-F – Screening for Autism in Females, SRS-2 – Social Responsiveness Scale, Second Edition

## Abstract

Autism Spectrum Disorder (ASD) in adult women without intellectual or language impairments is frequently under-recognized, due to subtler manifestations, greater use of compensatory social strategies, and reliance on diagnostic frameworks developed from male presentations. Diagnostic overshadowing, where autistic traits are misattributed to other psychiatric conditions, further delays accurate identification. This narrative review aims to critically evaluate recent evidence on the diagnostic challenges of ASD in adult women without intellectual or language impairments, assess the performance of widely used screening tools, and present recommendations for improving gender-sensitive diagnostic practices. A structured literature search was applied (PubMed, PsycINFO, Scopus; January 2010–July 2025; English language) targeting studies on females aged ≥18 years without intellectual or language impairment. Diagnostic accuracy, screening tools, camouflaging, misdiagnosis, and psychosocial outcomes were examined. Original research, meta-analyses, and systematic reviews were included, and a narrative synthesis approach was chosen due to study heterogeneity. Female-typical presentations often include subtle social-communication differences, context-specific restricted interests, and higher camouflage levels than males, which decrease the sensitivity of standard screening tools. Women are more likely to receive prior psychiatric diagnoses before ASD is recognized, contributing to mental health burdens and poorer functional outcomes. Current adult ASD screening tools have limited capacity to detect female phenotypes. Integrating camouflaging assessment, nuanced developmental histories, and updated, gender-inclusive screening instruments is essential to improving diagnostic equity.

## INTRODUCTION

Autism spectrum disorder (ASD) is a neurodevelopmental condition that has gained more attention in the past decade, partly because of the high social media focus on this topic [1] and due to the increasing number of adults seeking diagnosis later in life [2]. Despite the growing amount of information available, clinicians still face many challenges when diagnosing ASD in adults, especially women, not only because the diaganosis criteria have continuously evolved over the past 20 years [3], but also because most studies and guidelines focus on male presentation [4], as the reported prevalence is higher in men (2.75:1) [5].

Since the publication of the Diagnostic and Statistical Manual of Mental Disorders, Fifth Edition (DSM-5) in 2013 and Diagnostic and Statistical Manual of Mental Disorders, Fifth Editon, Text Revision (DSM-5-TR) published in 2022, diagnostic frameworks have increasingly recognized variability in presentation across sex/gender and culture, addressing some limitations of earlier male-centric criteria [6,7]. Despite these refinements, adult women without intellectual or language impairments remain at risk of under-identification, owing to subtler symptom expression, socially normative restricted interests, and the frequent use of compensatory behaviors such as camouflaging [8-11].

Camouflaging strategies, including learned social scripts, mimicry of neurotypical peers, and rehearsed conversational responses, can mask observable autistic traits during brief assessments, potentially lowering scores on screening instruments and delaying formal diagnosis [11,12]. Such strategies, hence adaptive in specific contexts, are associated with increased psychological distress, burnout, and delayed access to autism-specific mental health services. Misdiagnosis is common, with many women first receiving psychiatric labels such as anxiety disorders, depression, borderline personality disorder (BPD), or eating disorders before autism is considered by clinicians [13-17].

These diagnostic detours can result in prolonged periods without appropriate intervention, which contribute to cumulative functional disadvantages and increased mental health risks. Recent research indicates that women may require more extensive clinical interviews and developmental histories to accurately identify autism, especially when standardized screening tools are used in isolation [8-10,18-20]. Furthermore, cultural expectations regarding gendered behavior can obscure recognition of autistic traits in women, both for clinicians and for the individuals themselves [21].

The under-identification of ASD in adult women is not only a clinical challenge but also a public health concern, given the associated risks of unemployment, social isolation, and co-occurring mental health conditions [22-28]. Timely diagnosis can enable access to targeted support services, improve quality of life, and reduce the likelihood of secondary psychiatric morbidity. Addressing these diagnostic disparities requires integrating emerging evidence on female autism phenotypes into both clinical practice and research methodologies.

This narrative review synthesizes current evidence on how autism presents in adult women without intellectual or language impairments, examines the limits of commonly used screening tools in detecting female-typical traits, and explores the psychosocial effects of delayed or missed diagnoses. It offers evidence-based recommendations to improve diagnostic fairness through updated assessment tools, better clinician training, and increased awareness of gender-specific presentation patterns.

## MATERIAL AND METHODS

This narrative review examines diagnostic challenges of ASD in adult women (≥18 years) without intellectual or language impairments. A targeted literature search was conducted in PubMed, PsycINFO, and Scopus (January 2010–July 2025). Searches used multiple combinations of keywords related to autism spectrum disorder, female presentation, diagnosis, screening, misdiagnosis, camouflaging, and masking, with Boolean operators adapted to each database. Additional terms were added as relevant to emerging themes, and the search was enhanced through hand-searching reference lists and citation tracking from key papers. The main inclusion criteria were peer-reviewed studies in English that focused on diagnostic features, assessment tools, or psychosocial impacts in adult women without intellectual or language impairments. Studies solely on children, adolescents, or adults with co-occurring intellectual disabilities, and those lacking empirical data, were excluded. Although the focus was on recent research, earlier studies were included to contextualize the evolution of diagnostic criteria and to examine the continued use of screening tools developed under earlier diagnostic manuals. In cases where adult-specific evidence was insufficient, selected studies on children or adolescents were cited if their findings were judged relevant to adult female presentations. Given the heterogeneity of study designs, findings were integrated using a narrative synthesis that considered methodological type and quality rather than applying a formal systematic review protocol.

## TERMINOLOGY AND CONCEPTUAL FRAMEWORK

The terms Asperger Syndrome (AS) and High-Functioning Autism (HFA) have been used interchangeably for many years, before DSM-5 introduced the term ASD and the specifier 'with/without intellectual/language impairment'. They are still widely used in scientific papers, along with interchangeably used terms like 'impairment', 'delay', 'deficit', and 'disability'. All these changes obstruct the development of systematic reviews and meta-analyses because the search process becomes difficult due to the numerous possible keyword combinations.

Another obstacle in gathering and systemizing data is that most scientific literature is focused on children and adolescents, and most of them exclude women or mention them scarcely. A systematic review published in 2020 states that 92% of intervention trial participants were under 18 [29]. A 2024 narrative review emphasizes the continued exclusion of adults—particularly women—from neuroimaging studies on autism because of biased recruitment methods and male-centered diagnostic practices and criteria. It notes that nearly 70% of these studies include only male participants or very few female subjects, reinforcing cognitive neuroscience models based on male-centric data and contributing to the continued exclusion of both females and adults from autism research and diagnosis [30]. Longitudinal research tracking autistic individuals from childhood through adulthood remains scarce and often constrained by small sample sizes, limiting our understanding of developmental trajectories across the lifespan [31].

Over the last 5 years, scholarly attention to the 'female autism phenotype' and sex-related diagnostic bias in autistic adults—particularly women without intellectual or language impairment—has expanded noticeably, with newer studies concluding on outdated diagnosis methods and the necessity for new therapeutic approaches [13] as autism in men presents with more communication and social difficulties, while women present more cognitive disturbances and behavioral problems [16].

To avoid further confusion, this narrative review focuses on the diagnostic difficulties of ASD in adult women (aged 18 and over) without intellectual and language impairment using the terminology of the DSM-5-TR, the latest published diagnostic manual.

The conceptual framework considered the medical model versus the social model, neurodivergence as a disability versus neurodiversity as an identity, because autism has long been viewed negatively and stigmatized. The authors of this paper adopt a neurodiversity-informed perspective in alignment with the preferences expressed by many autistic individuals. We choose to use identity-first language ("autistic person") instead of person-first terminology ("person with autism"), as autism is often perceived as an integral part of an individual's identity [32]. While trying to respect the views of the autistic community, which frames autism as a variation in human experience, rather than a pathological condition, the term "autism spectrum disorder" will still be mentioned when referring to diagnostic criteria and protocols.

## EVOLUTION OF DIAGNOSTIC CRITERIA

ASD is a neurodevelopmental disorder that impacts social communication in individuals with restricted interests, repetitive behaviors, and a strong adherence to routine. The latest diagnostic criteria cited from the DSM-5-TR (2022) emphasize two main domains: social communication and interaction, and restrictive and repetitive behavior [6,33,34]. Initially, autism was seen as a diagnosis related to early childhood and associated with mental disability, beginning with the first use of the term in the Diagnostic and Statistical Manual of Mental Disorders, Third Edition (DSM-III) [35]. Over time, as the newer diagnostic manuals were published, the diagnostic criteria for autism underwent significant changes, as shown in Table 1. Despite this, aspects such as the development of symptoms from childhood to adulthood, high individual variability, gender differences, and cultural and racial differences were first mentioned very late, in 2022, in DSM-5-TR [7,34]. The last edition of the International Classification of Diseases (ICD) 11, updated in 2022, aligns with the DSM-5 criteria (2013) and lacks clear gender differentiation [7,36].

**Table 1:** Autism diagnostic criteria and gender inclusion timeline

Diagnosis Manual	Year of introduction	Key changes	Implications for women
DSM-III [35]	1980	Introduces the term "Infantile Autism", with rigid criteria focused on a male-centered approach, with a 30-month age limit for diagnosis.	Male-centered model; high risk of underdiagnosis in girls.
DSM-III-R [37]	1987	Removes the 30-month age limit and introduces the general category Pervasive Developmental Disorder (PDD).	Lack of gender differentiation persists.
ICD-10 [38]	1993	Use the term PDD, similar to DSM-III-R.	Outdated criteria, not adapted to gender differences.
DSM-IV [39]	1994	Defines subtypes: Autism, Asperger's, PDD-NOS. Focus on 3 main areas: socialization, communication, and repetitive behaviors.	Subtypes do not reflect the female phenotype; there is a risk of mislabeling or omission.
DSM-IV-TR [40]	2000	Text revision only. Retains DSM-IV structure. Adds clarifications and examples, but there are no major changes in diagnostic criteria.	Maintains subtype structure; female-specific traits are still overlooked.
DSM-5 [6]	2013	Removes subtypes, introduces ASD as a unified spectrum with 2 core criteria and 3 levels of severity. Recognize autism as a lifelong condition.	Masks gender differences; standard tools often miss subtle symptoms in women.
DSM-5-TR [7]	2022	Includes more diverse examples and the first reference to female phenotypes and camouflaging behaviors.	Opens the discussion on gender bias, but the contribution remains limited.
ICD-11 [36]	2022	Aligns with DSM-5, emphasizes spectrum and adaptive functioning, but lacks clear gender differentiation.	Lack of specific tools for identifying the female phenotype persists.

Note: DSM–III, Diagnostic and Statistical Manual of Mental Disorders, Third Edition; DSM-III-R, Diagnostic and Statistical Manual of Mental Disorders, Third Edition, Revised; ICD-10, International statistical classification of diseases and related health problems; DSM-IV, Diagnostic and Statistical Manual of Mental Disorders, Fourth Edition; DSM-IV-TR, Diagnostic and Statistical Manual of Mental Disorders, Fourth Edition, Text Revision; DSM 5, Diagnostic and Statistical Manual of Mental Disorders, Fifth Edition; DSM-5-TR, Diagnostic and Statistical Manual of Mental Disorders, Fifth Edition, Text Revision; ICD-11, International classification of diseases for mortality and morbidity statistics, 11^th^ revision.

The evolution of changes and the late recognition of gender differences affect both researchers and clinicians, as most rely on historical frameworks and symptom profiles mainly based on studies of men. This reinforces a male-centered view of autism that overlooks female-specific signs and results in ongoing underdiagnosis in women [16,32].

**Figure 1:**
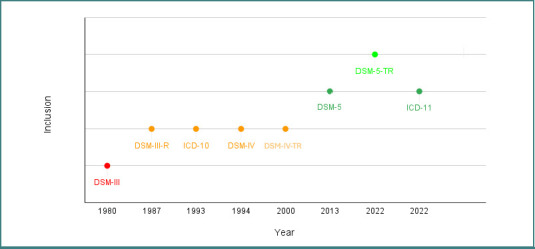
Timeline of ASD diagnostic inclusion across classification systems. Red – Diagnosis restricted to children under 30 months; Orange – Age limit removed, but child-focused (up to 18 years); Dark Green – Diagnostic criteria extended to adults; Light Green – First mention of female-specific phenotypes

Figure 1 illustrates key milestones in the inclusion and reconceptualization of ASD within major diagnostic manuals (DSM and ICD), starting from DSM-III (1980) and ending with DSM-5-TR and ICD-11 (2022) [6,7,35-40]. The timeline reflects changes in the formal recognition, categorization, and diagnostic criteria of autism, providing essential context for understanding how diagnostic frameworks have historically limited the identification of autistic adults—particularly women without intellectual or language impairments.

## INCREASED VISIBILITY, RECOGNITION, AND AWARENESS

In recent years, increased visibility and recognition of autism, driven by expanding research and the influence of social media, have contributed to broader public awareness and more inclusive narratives. This is complemented by a growing scholarly focus on autism spectrum disorder in women that aims to address historical diagnostic biases and better understand sex-specific presentations [30,32,41]. As the topic of autism has gained increased attention in scientific circles and social media, more adults have started seeking assessments, suspecting they might have undiagnosed autism, especially ASD without intellectual and language impairments. The symptoms in this latter group can easily go unnoticed over the years, even though these individuals report significant impairment in daily functioning. Because they have developed many masking strategies over time, they often are not diagnosed until later in life, as their intellect and language ability are sufficiently developed to ensure adaptation to society until specific stressors or higher professional or social expectations diminish the individual’s ability to cope, thus leading to a psychiatric evaluation [34,42].

Recent research highlights a significant rise in the diagnosis and recognition of ASD in adults and females, groups that have traditionally been underdiagnosed due to diagnostic biases and limited understanding of atypical symptom presentations. A large-scale cross-sectional study utilizing U.S. health records from 2011 to 2022 showed a 450% increase in autism diagnoses among adults aged 26–34, with a notable 315% increase among adult females during the same timeframe [43]. This trend points to growing awareness of adult autism and a narrowing gender gap in diagnostic rates, possibly due to shifting clinical practices and improved access to assessments. Complementing this, a narrative review regarding diagnostic disparities affecting girls and women with autism emphasizes that many women remain undiagnosed or misdiagnosed because of camouflaging behaviors (such as masking symptoms) and a prevailing male-centered diagnostic model. The authors advocate for gender-sensitive diagnostic tools and approaches that can more accurately capture the female autism phenotype [32]. A systematic review published in 2024 provides further insight regarding individuals diagnosed with autism in adulthood, often referred to as "late diagnoses”, which highlights how diagnostic delays are widespread in women, mainly due to societal and clinical misconceptions and the internalized masking of autistic traits. The study calls for enhanced clinician training and public education to facilitate timely and accurate adult diagnosis [44].

Together, these studies reflect a shifting diagnostic landscape, with greater attention now paid to how autism presents outside of childhood and outside of stereotypical male traits. The increase in diagnoses among adult women suggests both growing visibility and the need for continued efforts to dismantle gender and age-based diagnostic barriers.

## GENDER BIASED DIAGNOSIS

Gender-based diagnostic disparities in ASD persist, with women often being underdiagnosed or misdiagnosed due to historically male-centered diagnostic criteria and the increased likelihood of social camouflaging among autistic women, which contributes to delayed or missed recognition compared to their male counterparts [32,44]. The experiences and personal insights of 14 adult women who were diagnosed late with ASD were documented in a 2016 qualitative study to highlight the numerous challenges they face due to being misunderstood by clinicians and errors during the diagnostic process [45]. These findings were replicated in 2025, these two being the only in-depth analyses of interviews and personal insights into ASD without intellectual and language impairment in women to date [46]. These studies show that gender bias is prominent in both society and clinical settings, as teachers underreport behavioral and social problems of girls with autism, and later, clinicians underestimate ASD in women by requiring more criteria and more behavioral disturbances to make a diagnosis, compared to men. Camouflaging causes women to show fewer externalizing behaviors, leading first to internalization of stressors, manifesting later as anxiety, depression, trauma-related disorders, and sometimes eating disorders. They also score lower than their male counterparts on screening questions regarding restrictive and repetitive behaviors and interests [45,46], which subsequently causes clinicians to disregard this criterion in their diagnosis checklist, thus excluding an ASD diagnosis.

## MALE-CENTERED SCREENING AND DIAGNOSIS TOOLS

Autism was first described by Kanner in 1943 and by Asperger in 1944 as a condition similar to schizophrenia. Both authors observed behavioral patterns in male children (e.g., communication problems, social isolation, restrictive behaviors, stereotypes, echolalia) without mentioning descriptions of female presentation [33]. Subsequently, all studies focused on male predominance of autism as the researchers considered autism to be a male-specific disorder, focusing on the higher prevalence of ASD in men. This was explained by the belief that men’s brains are more focused on the concrete, more logical, and less prone to experience empathy, proposing the “extreme male brain theory” [47]. These facts have since been challenged by the studies summarized in Table 2, which show that the prevalence of autism in girls and women is higher than previously estimated [48-52].

**Table 2:** Prevalence of autism, male-to-female ratio

Estimated male-to-female ratio	Population Method	Key Findings	Study
4:1 (traditional estimate)	Clinical diagnosis, DSM-IV/DSM-5 tools	Longstanding average based on male-centric diagnostic criteria. Likely underestimates women.	Lai & Szatmari (2020) [48], Loomes *et al*. (2017) [49]
3:1	Community-based samples (US and UK)	With better tools and awareness, the gap narrows. More girls are being identified.	Lai *et al*. (2015) [50], Grosvenor *et al*. (2024) [43]
2:1	Broader screening, adult self-reports	Studies using broader inclusion criteria and self-identification show higher rates in women.	Napolitano *et al*. (2022) [51]
1:1.2 (F>M)	Simulation modeling correcting for bias	A modeling study by McCrossin suggests the actual prevalence in women may even exceed men when adjusting for diagnostic bias.	McCrossin (2022) [52]

Note: US, United States; UK, United Kingdom

Over the years, many screening and diagnostic tools have been created, primarily targeting children and individuals with intellectual and language impairments. Only a handful of tools for adults have been updated to align with the latest diagnostic manuals (DSM-5-TR and ICD-11). Furthermore, even fewer of these are calibrated for gender differences. Table 3 [18,53-63] summarizes the existing screening and diagnostic tools.

**Table 3:** Screening and diagnostic tools for ASD

Scale	Authors	Year	No. of items	Limitations	Gaps
AQ 50	Baron-Cohen *et al*. [18]	2001	50	No gender calibration, but women are shown to report lower scores.	Self-report, no retroactive symptom check; DSM IV-based.
RAADS-R	Ritvo *et al*. [53]	2011	80	No gender calibration.	Self-report, no retroactive symptom check. DSM-IV-based
ADI-R	Lord *et al*. [54]	1994	93	No gender calibration, focused on children.	Focusing on retroactive symptoms might not capture adult-specific phenotype. DSM-IV-based.
ADOS-2	Lord *et al*. [55], module 4 revised by Hus & Lord [56]	2012 / 2014	29-31	No gender specific, women can go under the radar.	Golden standard, constructed with a male brain in mind. DSM-IV-based but revised for DSM-5.
SRS-2-Adult Self-Report Form	Constantino and Gruber [57], revised by Bruni [58]	2012 / 2014	65	Not calibrated for women.	Not very sensitive, it might indicate a false negative. DSM-IV-based but revised for DSM-5.
SfA-F	Benevides [59], by Marques in Brazil [60]	2024 /2025	34	Designed specifically for women. Limited studies.	Self-report, not enough research. The only diagnosis tool based on DSM-5 TR. Still under development.
GQ-ASC	Brown *et al*. [61]	2018	58	Calibrated for girls. Limited studies.	Self-report, not very sensitive in detecting the camouflage. DSM-5 based.
CAT-Q	Hull *et al*. [62]	2019	25	Targets the camouflage traits for girls.	Complementary tool, not screening or diagnosis.
BASC-3	Reynolds & Kamphaus [63]	2015	160-176	Some versions can go up to age 25.	Not specifically for autism, but for behavioral disorders.

Note: AQ-50, Autism-Spectrum Quotient, 50 items version; RAADS-R, Ritvo Autism Asperger Diagnostic Scale-Revised; ADI-R, Autism Diagnostic Interview-Revised; ADOS-2, Autism Diagnostic Observation Schedule, Second Edition, Module 4; SRS-2, Social Responsiveness Scale, Second Edition, Adult Self-Report Form; SfA-F, Screening for Autism in Females; GQ-ASC, Girls Questionnaire for Autism Spectrum Condition; CAT-Q, Camouflaging Autistic Traits Questionnaire; BASC-3, Behavior Assessment System for Children, Third Edition.

The tools presented above are male-centric as they were developed and normed on predominantly male samples, shaping item content around a 'male' presentation, and show measurement non-invariance (items functioning differently by sex), which reduces sensitivity to female/gender-diverse phenotypes [64-66]. In parallel, women and some gender-diverse people more often camouflage/mask autistic traits, systematically lowering scores on items that rely on overt social-communication differences and stereotyped behaviors, thereby delaying or obscuring case detection [11,67,68]. Contemporary reviews further conclude that current procedures and instruments insufficiently capture the female autism phenotype, contributing to later or missed diagnoses and calling for sex/gender-aware adaptation and re-norming of instruments such as AQ and Ritvo Autism Asperger Diagnostic Scale-Revised (RAADS-R) [32,51,69].

Recent developments in female-specific autism screening have introduced new tools to improve detection accuracy. The Girls Questionnaire for Autism Spectrum Condition (GQ-ASC) has demonstrated that it correctly identifies approximately 80% of cases [61]. At the same time, the Screening for Autism in Females (SfA-F) measure is a new instrument specifically designed to capture female-specific presentations of autism [59,60]. The Camouflaging Autistic Traits Questionnaire (CATQ) is a supplementary tool targeting camouflaging in verbally fluent autistic adults [62]. While these instruments show promise for identifying autism in women, current evidence remains limited, and further validation across diverse populations is required to establish their reliability and clinical utility.

## CLINICAL PRESENTATION AND DIAGNOSIS BARRIERS IN AUTISTIC WOMEN

### Female phenotype

Autism in women and girls presents differently compared to men due to differences in social communication, less restrictive interests, and masking behaviors. The female autism phenotype is not widely recognized by clinicians, leading to delays in referral, diagnosis, and adequate interventions and support, as current diagnosis criteria are primarily based on the male phenotype, with diagnosis thresholds between females and males being similar [8-10].

Compared with the male-typical profile, autistic girls and women often show more superficially typical social behavior, staying near peers, weaving in and out of groups, and using compensatory strategies that mask difficulties. In contrast, autistic boys more often appear solitary or disengaged [70]. In communication, girls may present 'linguistic camouflage', producing more typical-sounding filled-pause patterns that make speech seem socially typical despite similar underlying social-pragmatic challenges [71]. In restricted and repetitive behaviors/interests, women tend to show fewer overt restricted interests, and their interests are more often age- and gender-congruent (e.g., animals, fiction, celebrities), which can be overlooked in assessments tuned to male-typical themes and mannerisms [72].

A 2024 meta-analysis further indicates that, on standard measures, men show greater observable social-interaction difficulties, while women show relatively greater cognitive/behavioral challenges, patterns consistent with camouflaging and phenotype differences that bias current tools toward male presentations [16].

Research on the differential diagnosis of autism in both genders confirms the biases experienced by clinicians. An original quantitative study using data from a longitudinal national registry of 1,019 autistic adults found that 62.7% of females and 37.0% of males had at least one prior psychiatric diagnosis. Comorbidities were more common in females (67.0% vs. 51.0%), most frequently mood disorders and anxiety. Women were also more likely to have a previous diagnosis that was not upheld, with rates of 47.0% compared to 27.3% in men [73]. More recent research confirms these findings, reporting that 25% of autistic adults had received at least one psychiatric misdiagnosis prior to their autism diagnosis, reinforcing evidence that such errors are common and may disproportionately affect women [13].

### Camouflaging and masking

In individuals with normal or above-average intelligence and well-developed language skills, autism symptoms can remain hidden until late in life. This is because they use coping strategies to follow a script, effectively 'acting like a neurotypical', and consciously learn how to speak, interpret non-verbal cues, and mimic social behaviors to fit into society. Unfortunately, this approach often carries a significant cognitive cost, which can lead to fatigue, burnout, and comorbidities such as depression and anxiety [11]. Women more often use camouflaging than men due to different gendered expectations and societal standards [11,74], which can lead to misdiagnosis and delays because they seek evaluation only when they can no longer compensate [12]. The masking of autism symptoms in women might be explained by a higher capacity to integrate social stimuli and by the reduced frequency of restrictive repetitive behaviors. Girls may quickly learn how to mimic typical behavior in social situations and often have better language skills, while language delay is more common in males and is a primary reason parents seek medical services [11,75].

Self-reported and meta-analytic studies consistently find higher CAT-Q scores among autistic women than men, evidencing greater masking (meta-analyses of CAT-Q) and linking camouflaging with later age at diagnosis in females [76,77].

### Comorbidities

Comorbidities are common in ASD, with 70-80% of diagnosed adults having at least one secondary condition [22], and the comorbidity burden is even higher in women [21]. The most common co-occurring conditions are attention-deficit hyperactivity disorder (ADHD), with an approximate lifetime prevalence of 40.2% [14], trauma-related conditions, and post-traumatic stress disorder (PTSD) [78], along with anxiety and depression, which have an approximate lifetime prevalence of 42%, respectively 37% [23], and obsessive-compulsive disorder (OCD) [15]. 65.8% of women had a previous psychiatric diagnosis, compared to 34.2% of men [13]. This is because clinicians are less likely to consider autism as a diagnosis pathway in women compared to men, with more focus being placed on symptoms of related comorbidities like mood disturbances, trauma-related symptoms, obsessions and compulsions, personality traits, anxiety, and behavior issues other than those linked to autism [16]. Women also tend to express more externalizing behavioral traits compared to men, symptoms like impulsivity, disordered eating, emotional instability, and self-harm being more frequent, leading to diagnoses like anorexia nervosa and borderline personality disorder (BPD). These conditions often coexist with autism, but the autistic traits, especially when they are more subtle, frequently go unnoticed [17].

Women with ASD are proven to be more vulnerable to manipulation and abuse, as well as to the accumulation of comorbidities and symptoms indicative of other conditions like PTSD, OCD, and BPD, which can mask ASD symptoms even more and allow them to go "under the radar" [45,46,79].

### Late diagnosis and misdiagnosis

ASD is a condition that significantly impacts individual development and well-being [11]. The earlier the diagnosis, the better the outcome due to earlier access to adequate therapy and earlier support, resulting in greater functionality later in life [80]. Autism is a complex disorder, and its diagnosis requires an experienced clinician who has to consider many variables. One of the most important aspects is the history and persistence of the symptoms, as they tend to appear prominently from early childhood and can be present even in toddlers (e.g., lack of visual contact, reduced facial expressiveness, and gestural communication) [81]. This is why interviewing a parent or primary caregiver who can provide a detailed account of early developmental years is crucial, but it can be challenging to do in the case of adult patients [82].

Misdiagnosis can cause social isolation and self-stigma, worsening the burden of pathology and dysfunction in individuals who, if properly diagnosed, would have had much better outcomes [13]. Women encounter more obstacles in getting diagnosed than men, mainly because of the still-persistent belief in the "extreme male brain" theory of autism, which many clinicians still adopt [16]. This leads to a lack of flexibility during assessments and seeing better social skills and fewer restrictive behaviors and interests in women as reasons to rule out an ASD diagnosis. Female children, even if they display fewer symptoms than boys, face the same communication challenges and functional impairments. Due to a lack of support, these issues become internalized, leading to later manifestations and symptoms during stressful times or in response to external factors, which require clinical attention and often result in a late diagnosis [79].

### Psychological and functional impact of late diagnosis

A late diagnosis can have a significant impact. The person diagnosed not only feels relief after many years of misunderstanding but also experiences grief over the time spent without a confirmed diagnosis following the ASD diagnosis. [73].

A delayed, missed, or incorrect diagnosis significantly affects the individual and society. The emotional and mental health consequences range from increased anxiety and depression to feelings of isolation and low self-esteem. The reported social and interpersonal challenges are even greater for women, as they experience emotional exhaustion and burnout after years of sustained effort to hide autistic traits [83], leading to higher costs for mental health services as symptoms worsen due to delayed access to proper interventions caused by misdiagnosis and inappropriate treatment [45]. The educational and occupational consequences are also significant, as delayed diagnosis results in missed opportunities for early intervention, which can later negatively impact academic achievement and social skills development [24]. Late-diagnosed students are often misunderstood by the educational system and their peers, leading to bullying, social exclusion, and unmet educational needs [25]. Adults diagnosed late often face challenges at work, resulting in extended leaves of absence due to social isolation, sensory issues, and executive function problems [25]. These issues can later lead to unemployment or underemployment [26]. Lack of diagnosis also delays access to academic and workplace accommodation, worsening professional outcomes, and later affecting overall functionality [27]. This has been confirmed by a longitudinal study conducted over 8 years in the Netherlands, which followed a cohort of 2,449 autistic adults (1,077 men, 1,352 women, and 20 non-binary individuals) and also identified factors associated with stable employment for individuals with ASD, such as lower severity of autistic traits, higher education, and earlier ASD diagnosis [28].

Given the intense feeling of defectivity, impaired social adaptation, and lack of professional symptom recognition and support, most women appeal to online resources and self-diagnose, a fact that leads later to social stigma as the diagnosis is often not officially recognized. Social exclusion causes many women to engage in risky behaviors such as self-harm and substance abuse, which can lead to a diagnosis of BPD, and often a comorbidity of autism in women. However, the failure to recognize autistic traits beneath these behaviors results in incorrect therapeutic approaches and worsens the prognosis [84].

### Gaps in screening and diagnosis tools

The AQ is one of the most widely utilized screening instruments due to its free availability. It was developed based on DSM-IV-TR criteria, which are predominantly male-centric. It includes a *social skills* subscale that reflects ease in social situations, warmth in interaction, and preference for groups [18]. However, evidence shows this subscale has critical limitations in detecting autism in women. Autistic female patients frequently mask autistic traits, practicing social scripts, mimicking others, and sustaining eye contact, which inflate their performance on self-reported questions about social confidence and friendliness. These compensatory strategies suppress the manifestation of deeper social struggle, which the AQ does not detect [85].

Recent studies show that the AQ Social Skills items do not function equivalently across sexes. Their measurement invariance analysis indicated gender bias at the item level. While females often report higher empathy or friendship motivation, their internal experiences of cognitive load, anxiety, and autistic burnout remain hidden behind “social success” [64].

Systematic reviews show that standardized narrow measures of social communication and interaction, mirrored in the AQ Social Skills and Communication subscales, are less sensitive to nuances in female autistic presentation [86].

The NICE guidelines identify the ADOS-2 as a gold-standard observational tool for autism assessment within comprehensive diagnostic pathways [87]; however, evidence suggests it may be less sensitive to female presentations. ADOS-based confirmation disproportionately excludes autistic women from research samples [88], and item-level analyses indicate alignment with male-typical profiles [89].

## RESEARCH GAPS IN THE CURRENT LITERATURE

Despite growing attention, key evidence gaps persist for autistic women without intellectual or language impairment. Diagnostic research still relies on male-centric instruments, with item bias documented on the AQ; only a minority of items show gender invariance, risking under-identification in women [64]. Although recognition is improving, age at diagnosis remains higher for females, and trajectories of late-identified women are poorly described [90]. Large surveillance datasets confirm uneven identification by sex and race/ethnicity but provide limited adult follow-up needed to link early disparities to later outcomes [91,92]. Evidence confirms sex/gender differences in presentation, yet findings on restricted and repetitive behaviors remain inconsistent, reflecting measurement and construct gaps between genders [86,93].

There is no consensus operationalization of masking and camouflaging in clinical workflows, and few studies test whether incorporating camouflaging metrics reduces missed diagnoses in women [94]. Intervention research remains sparse for verbally fluent adult women; guidance is primarily extrapolated from mixed-sex samples or expert opinion, with limited trials tailored to female priorities [95]. Finally, intersectional factors, culture, race, and socioeconomic status, are acknowledged as drivers of inequity. Still, prospective, adequately powered studies connecting these factors to diagnostic timing and mental-health outcomes in adult women are rare [95]. Collectively, these gaps impede accurate screening, prompt diagnosis, and specific support for this subgroup. Few rigorously evaluated interventions focus specifically on adult autistic women or tailor their content to meet female-salient needs, resulting in a notably limited evidence base for both effectiveness and implementation [96]. Existing research also highlights an elevated risk of sexual and physical victimization among autistic women [97]; however, prevention programs and trauma-informed care approaches tailored to this population remain largely absent. Furthermore, equity and intersectionality are rarely addressed, with limited investigation into how outcomes vary by race, ethnicity, socioeconomic status, LGBTQIA+ identity, or cultural background [98-100], gaps that constrain the generalizability of findings and hinder the design of responsive, inclusive services.

## INTERSECTIONALITY – MINORITIES AND IDENTITY

For autistic women without intellectual and language impairments, intersecting identities can lead to diagnostic delay and shape psychosocial outcomes. Cultural norms around femininity, gender roles, and sociability may encourage camouflaging and discourage help-seeking, leading clinicians to overlook autistic traits when surface social behavior appears typical [90]. In some communities, stigma surrounding neurodevelopmental labels further suppresses disclosure and limits family advocacy, delaying referral until crises emerge or comorbidities accumulate [101,102]. Race and ethnicity also influence pathways to care: US surveillance data continue to show uneven identification by race/ethnicity despite overall gains, indicating that autistic girls of color are at heightened risk for later or missed diagnoses [91,92]. Such delays are associated with greater internalizing symptoms, burnout, and reduced access to tailored support in adolescence and adulthood, further increasing the personal and societal burden. Camouflaging impedes diagnostic recognition and delays treatment, amplifying feelings of thwarted belonging, elevating the risk of internalizing symptoms, and increasing suicidality [103].

Socioeconomic status intersects with the factors mentioned above by constraining access to specialist evaluation, continuity of care, and culturally responsive therapy; lower-income women face longer wait times, fewer provider options, and higher out-of-pocket burdens that perpetuate unmet mental-health needs [103]. Finally, higher rates of LGBTQIA+ identity among autistic women create additional minority stress that can intensify anxiety and depression when services are not affirming [104,105].

Addressing intersectional inequities requires a culturally competent assessment, proactive screening in underserved settings, and neurodiversity-affirming mental-health care.

## FUTURE DIRECTIONS AND EMERGING APPROACHES

Table 4 summarizes recent advances and recommendations to enhance diagnosis, assessment, and therapeutic support for autistic women without intellectual or language impairment. A personalized approach is preferred, and interviews with parents and caregivers should be conducted, when possible, to obtain data regarding childhood behaviors and neurodevelopmental history.

**Table 4:** Gender-sensitive autism assessment and support: key recommendations

Recommendation	Domain	Author(s), Year
Broaden diagnostic assessments to include female-typical exemplar behaviors, masking, and scripting. Include checklist-style queries oriented toward social camouflage.	Diagnosis & screening	Cook, 2024 [32]
Use gender-sensitive diagnostic criteria, ensuring social communication tools capture subtle social distress despite surface competence.	Screening	Lai *et al*., 2023 [95]
Clinician consensus recommends integrating female-normed interview prompts and item-level recalibration in standard assessments.	Diagnosis protocol	Trundle *et al*., 2025 [106]
Prioritize self-report and qualitative histories from autistic women to counter diagnostic overshadowing and clinician bias.	Diagnostic process	Hull *et al*., 2017[83], Hull *et al*., 2020 [67]
Introduce neurodiversity-affirming therapy frameworks, emphasizing strengths and reducing masking load; avoid deficit-based approaches.	Therapy	Lai *et al*., 2023 [95]; Lerner *et al*. 2023 [107] Khudiakova *et al*., 2024 [108]
For high-verbal, intellectually able women, promote mindfulness-based interventions and mental health support rather than strict behavioral training.	Therapy & support	Cook *et al*., 2021 [94]; Agius *et al*., 2024 [109]; Forbes *et al*., 2023 [110];

## DISCUSSION AND LIMITATIONS

Recent evidence underscores the necessity of developing gender-sensitive diagnostic tools capable of accurately identifying the female autism phenotype, which may manifest through subtler social communication differences and heightened use of camouflaging strategies [82]. Since the presentation of autism in women remains insufficiently recognized by educators and clinicians, targeted training on the early signs of ASD in women is essential for increasing awareness of gender-specific manifestations. This can help reduce delays in diagnosis [111] and lessen long-term societal costs. Core parts of a thorough diagnostic process, such as qualitative assessments, developmental history, standardized diagnostic criteria, and psychometric evaluation, must be consistently incorporated into clinical practice. As diagnostic frameworks and terminology evolve, sustained specialist training and intersectional research are essential. Furthermore, prospective and retrospective quantitative studies should be undertaken across diverse populations and gender identities, beyond the binary categories of male and female [8,58], to advance understanding of clinical presentation, developmental trajectory, outcomes, and comorbidities. Research on ASD in adults, especially women and gender-diverse individuals, remains limited and fragmented, hindered by shifts in terminology that make systematic reviews and meta-analyses more difficult. This lack of clarity leads to confusion among clinicians and the general public about appropriate diagnostic language. Increasing research into the lived experiences and challenges of autistic adults could help develop tailored vocational interventions, strengthen support networks, and guide improvements in mental health policy [28].

The current narrative review is limited by the diverse nature of the available literature and the lack of studies focusing specifically on autistic women without cognitive or language impairments. Differences in study design, diagnostic criteria, and terminology further hinder the comparability of findings and limit the ability to make broad conclusions. These issues are not unique to research on autistic women; studies on autistic adults in general face similar challenges, including variability in sampling methods, reliance on cross-sectional designs, and frequent dependence on self-reported diagnoses. Another limitation of the present review is the absence of specific consideration for the experiences and perspectives of sexual and cultural minority groups.

## CONCLUSION

Autistic women without cognitive or language impairments remain an underrecognized and underserved group, partly due to male-centered diagnostic frameworks and limited awareness of the female autism phenotype. Implementing updated, validated, and inclusive screening instruments, integrating developmental history and behavioral observation into assessment, and improving clinician training on female autism phenotypes are essential for diagnostic equity. Future research should prioritize intersectional approaches and explore neurobiological, cognitive, and social mechanisms underlying sex-related presentation differences to inform assessment practices and intervention strategies.
